# True Temperance

**Published:** 1876-05

**Authors:** 


### TRUE TEMPERANCE.

Mr. Editor—Your oft-repeated exhortations to moderation in eating, in your excellent Journal, and in private counsel, have been of inestimable value to me. Is it not a sad fact that men's ideas of temperance, for the most part, respect *drinking* alone? I submit the question, without attempting an answer, whether the modern rage of total abstinence from drink has not incidentally done evil in propagating this false and pernicious notion. It is believed that your Journal, and books and papers of a similar character, are bringing men's minds up to larger views. How many are there whose consciences would scarcely let them take wine sauce for a pudding, or season a mince-pie with a spoonful of brandy, who keep themselves in a constant condition of obesity. Emancipated from the bottle, they are in bondage to the platter! If the spectacle were not so mournful, it would be ridiculous!

As a rule to which there is almost never an exception, I find it best to rise from the table with a feeling of hunger—not a gnawing desire for food, but a keen appetite which would eagerly seize upon another plateful. I have found it desirable —I might almost say necessary—to treat myself just as I treat my horse, in the matter of food; measure out the quantity which judgment and conscience approve; and, after that is deliberately devoured, let the stomach cry out for more as it pleases, always meet it with an inexorable denial. A man who has been overeating all his life (as I have been till lately) can hardly trust himself at full liberty even over a plain meal, with more safety than he could to give his horse free access to the oat-bin. After the bondage to overeating is once broken in this stern discipline, self-denial becomes easier, the fury of a morbid appetite is calmed down, and the stomach becomes more rational in its demands. As for myself, however, though moderation at table is less difficult than it used to be, yet I have found that I am very much like a reformed drunkard as respects eating; and the least overstepping of the prescribed limit rouses up the old appetite again in all its original and untamed violence.

